# The effects of medicinal plants on renal function and blood pressure in diabetes mellitus

**DOI:** 10.5830/CVJA-2012-025

**Published:** 2012-09

**Authors:** CT Musabayane

**Affiliations:** Department of Human Physiology, Faculty of Medicine, University of KwaZulu-Natal, Durban, South Africa

**Keywords:** diabetes mellitus, diabetic nephropathy, medicinal plants, hypertension

## Abstract

**Abstract:**

Diabetes mellitus is one of the most common chronic global diseases affecting children and adolescents in both the developed and developing nations. The major types of diabetes mellitus are type 1 and type 2, the former arising from inadequate production of insulin due to pancreatic β-cell dysfunction, and the latter from reduced sensitivity to insulin in the target tissues and/or inadequate insulin secretion. Sustained hyperglycaemia is a common result of uncontrolled diabetes and, over time, can damage the heart, eyes, kidneys and nerves, mainly through deteriorating blood vessels supplying the organs. Microvascular (retinopathy and nephropathy) and macrovascular (atherosclerotic) disorders are the leading causes of morbidity and mortality in diabetic patients. Therefore, emphasis on diabetes care and management is on optimal blood glucose control to avert these adverse outcomes.

Studies have demonstrated that diabetic nephropathy is associated with increased cardiovascular mortality. In general, about one in three patients with diabetes develops end-stage renal disease (ESRD) which proceeds to diabetic nephropathy (DN), the principal cause of significant morbidity and mortality in diabetes. Hypertension, a well-established major risk factor for cardiovascular disease contributes to ESRD in diabetes. Clinical evidence suggests that there is no effective treatment for diabetic nephropathy and prevention of the progression of diabetic nephropathy. However, biomedical evidence indicates that some plant extracts have beneficial effects on certain processes associated with reduced renal function in diabetes mellitus. On the other hand, other plant extracts may be hazardous in diabetes, as reports indicate impairment of renal function. This article outlines therapeutic and pharmacological evidence supporting the potential of some medicinal plants to control or compensate for diabetes-associated complications, with particular emphasis on kidney function and hypertension.

## Abstract

Diabetes mellitus is a global disease affecting both the developed and developing nations. Epidemiological data suggest that at least one in 20 deaths are attributable to diabetes and related complications, a proportion which increases to at least one in 10 deaths in adults aged 35 to 64 years.^1^ The figure is considered to be an underestimate since most individuals die from cardiovascular and renal-related complications.^2^ World Health Organisation data show that the age-standardised death rate for diabetics in South Africa is 85 per 100 000. Death rates in other sub-Saharan African countries range from 21 to 49 per 100 000, compared with 18 in the USA and six per 100 000 in the UK.^3^

The principal causes of mortality in type 1 and 2 diabetes patients are disorders grouped as microvascular (retinopathy and nephropathy) and macrovascular (atherosclerotic) complications.^4^,^5^ Macrovascular diseases account for the majority of deaths in type 2 diabetes patients, and the presence of hypertension is associated with a four- to five-fold increase in mortality.^6^ A causal relationship between chronic hyperglycaemia and diabetic microvascular disease, long inferred from various animal and clinical studies,^7^ has now been established by data from the Diabetes Control and Complications Trial (DCCT) controlled clinical study.^8^

Conventional diabetes therapy using blood glucose-lowering agents such as sulphonylureas, insulin therapy, α-glucosidase inhibitors, peroxisome proliferator gamma (PPAR-γ) agonists and biguanides has limitations. For instance, insulin therapy does not achieve glycaemic control in patients with insulin resistance, and oral hypoglycaemic agents may lose their efficacy after prolonged use. Previous studies elsewhere suggest that insulin is not only ineffective in preventing type 1 diabetes in patients at risk of developing this condition, but it can also cause cardiovascular disease.^9^,^10^ Furthermore, conventional drugs are not easily accessible to the general population in developing countries due to socio-economic conditions.^11^,^12^ Hence there is an urgent need to find affordable treatments that are effective in slowing the progression of diabetic complications.

Traditional herbal medicine is used by many rural African communities to treat a range of diseases, including diabetes. Anecdotal evidence suggests that diabetic complications are less common in rural populations, attributable to either the beneficial effect of plant medicines or to the fact that other risk factors that aggravate diabetes in the urban context are less prevalent in rural situations. The World Health Organisation not only encourages the use of plant medicines, but also recommended scientific evaluation of the hypoglycaemic properties of plant extracts.^13^ Estimates indicate that more than 70% of the world’s population uses resources derived from traditional medicine to control diabetes.^14^ Medicinal-plant home remedies are used as crude extracts or standard, enriched fractions in pharmaceutical preparations.

Research summarised in a recent review^15^ showed that several southern African plant species used by rural communities as traditional medicines had hypoglycaemic effects in streptozotoc-ininduced (STZ) diabetic rat. Furthermore, some species had antihypertensive properties.^16^-^19^ The impact on the kidney varies, with some species being reno-protective, whereas others had a deleterious effect on kidney function. By identifying the bio-active compound, oleanolic acid (OA), which confers reno-protection, we have been able to demonstrate the effectiveness of this agent in STZ diabetic rats.

The focus of this article is to evaluate current evidence on plant extracts used for the management of hypertension and kidney disease in diabetes. The beneficial as well as deleterious effects of medicinal plants in both conditions are discussed based on reports on plants frequently used in the southern Africa setting. Herein, a medicinal plant is defined as any plant which provides health-promoting characteristics, temporary relief or has curative properties.

## Antihypertensive therapy and diabetic renal disease

Diabetic complications, which include damage to large and small blood vessels, can lead to coronary heart disease, stroke and hypertension, the latter being a well-established major risk factor for cardiovascular disease that contributes to end-stage renal disease (ESRD). Reduction of blood pressure (BP) is therefore an efficient way of preventing or slowing the progression of ESRD. Conventionally, reno-protection is achieved through reduction in BP with antihypertensive regimens.^20^-^23^ Several studies however document that antihypertensive treatment in diabetes not only improves the quality of life,^24^-^27^ but also reduces renal complications.^28^

The major antihypertensive drug classes widely used include thiazide diuretics, angiotensin converting enzyme (ACE) inhibitors, angiotensin receptor blockers (ARBs), β-blockers, central sympatholytic agents, calcium channel antagonists and other vasodilators. However, some antihypertensive agents, for example, thiazide diuretics and β-blockers deleteriously influence glycaemic control.^29^

To date, the most effective treatments for diabetic nephropathy (DN) are the antihypertensive drugs, particularly those that target the renin–angiotensin system (RAS) such as ACE inhibitors, angiotensin-1 receptor antagonists, or their combination.^25^,^30^,^31^ Although these treatments may retard the progressive decline in renal function in diabetes, clinical trials suggest that there is no effective treatment for DN.^8^

For these reasons, novel anti-diabetic therapeutic agents that supplement, substitute or complement the existing modern medications to ameliorate renal function in diabetes constitute novel therapeutic strategies for diabetes. Evidence from biomedical literature suggests that some plant extracts have protective effects against cardiovascular disease in diabetes.^32^ The following sections evaluate the therapeutic and pharmacological evidence for the use of some of the medicinal plants and their bioactive phytochemicals in cardio-renal related diabetic complications, as well as the potential for nephrotoxicity from other plant extracts.

## Natural plants for cardiovascular disease

Several plant extracts with potential therapeutic properties for the treatment of hypertension and complications such as coronary heart disease, angina, arrhythmias and congestive heart failure have been identified.^33^-^36^ Traditional medicinal healers in southern Africa have used *Helichrysum ceres* S Moore [Asteraceae] to treat kidney and cardio-respiratory disorders.^37^ Recent laboratory studies suggest that the hypotensive effects of *H ceres* leaf extract in anaesthetised male Sprague-Dawley rats could in part be attributed to the extract’s natriuretic and diuretic properties.^38^ We reported that *H ceres* ethanolic leaf extract’s hypotensive effects were elicited in part by the direct relaxant effects on cardiac and vascular smooth muscles.^39^ The data suggested that lowering of blood pressure was due to reduced peripheral resistance elicited by the extract’s vasodilatatory effects on the vascular smooth muscles, mediated in part via the endothelium-derived factors (EDRF). This suggestion was corroborated by the observations that *H ceres* leaf extract elicited potent negative inotropic and chronotropic effects *in vivo* and exhibited vasorelaxant effects in vascular tissue preparations.

We also reported that *Ekebergia capensis* Sparrm (Meliaceae) leaf extract prevented the development of hypertension in weanling genetically hypertensive Dahl salt-sensitive (DSS) rats, which develop hypertension as they age.^19^ The *in vivo* reduction in blood pressure by the extract occurred without significant alterations in the heart rate, suggesting that the *in vitro* cardiovascular effects of the extract significantly contributed to the hypotensive effects. Indeed, studies showed that the hypotensive effect of *E capensis* leaf extract was in part mediated via modulation of total peripheral resistance of the vascular smooth muscles, as evidenced by the extract’s elicited dose-dependent vasorelaxations in endothelium-intact and endothelium-denuded aortic ring preparations. It should be noted that lanoxin, one of the cardiac glycosides found in a number of plants, has specific effects on the myocardium.

## Kidney function changes in diabetes mellitus

Sustained hyperglycaemia is the main cause of the changes in kidney function in diabetes mellitus. Hyperglycaemia leads to the increased formation of advanced glycation end-products (AGEs), oxidative stress, activation of the polyol pathway and hexosamine flux, causing inflammation and renal damage.^40^ AGEs result in the increased production of extracellular matrix proteins in endothelial cells, mesangial cells and macrophages in the kidney.^41^ Additionally, AGEs have been shown to reduce matrix protein flexibility through cross-link formation of the extracellular matrix proteins, leading to an abnormal interaction with other matrix components.^41^

Irrespective of all the other structural and functional changes, the mesangial alterations appear to be the main cause of declining renal function in experimental diabetic animal models.^42^ For example, hyperfiltration, which occurs in the early stages of DN has been attributed to increased mesangial production of vascular permeability factors in response to stretching.^43^ The subsequent decline in glomerular filtration rate (GFR) as nephropathy progresses may be due to expansion of the mesangial matrix, which compresses the glomerular capillaries, thereby reducing the filtration surface area and impairing the mechanism that maintains the normal glomerular capillary hydrostatic pressure.^42^ The fall in GFR also reduces the sodium load delivered to the macula densa cells, resulting in enhanced tubulo-glomerular feedback (TGF).^44^ In turn angiotensin II production increases due to hyperactivation of the renin–angiotensin–aldosterone system,^45^ causing more reabsorption of sodium and an increase in systemic blood pressure.

The accumulation of AGEs can be prevented by antioxidants such as flavonoids or by preventing the glucose-dependent formation of intermediate products (Amadori, Schiff bases or Milliard products). Indeed, blocking or deleting AGEs’ receptor (RAGE) in experimental animals reversed atherosclerosis.^46^ Amino guanidine and pyridoxamine, AGEs formation inhibitors, had reno-protective effects in diabetic animals.^47^,^48^ Furthermore, inhibition of AGEs effects could be achieved through breaking of the AGEs cross links by drugs such as alagebrium or inhibition of AGE signal transduction.^48^

Tanaka *et al*.^49^ reported that the biguanide metformin, the only example of an approved antidiabetic from a herbal source, French lilac (*Galega officinalis*) may be useful in the prevention of the development of AGEs. The *Panax quinquefolium* (Linnaeus) [Araliaceae] extracts, a phyto-oestrogen derived from *Vitis vinifera* (Linnaeus) [Vitaceae] (resveratrol), curcumin from *Curcuma longa* (Linnaeus) [Zingiberaceae] and glycosides from *Stelechocarpus cauliflorus* (RE Fr) [Annonaceae] have also been reported to inhibit formation of AGEs or RAGE.^50^-^56^

## Diabetic nephropathy

Renal disease is a common and often severe complication of diabetes, with the majority of patients with 18 years’ duration showing signs of diabetic renal involvement.^57^ In general, about one in three patients with type 1 or 2 diabetes develops ESRD which proceeds to DN, the principal cause of significant morbidity and mortality in diabetes.^8^ The onset of DN is associated with a progressive rate of decline in renal function, urinary albumin excretion and glomerular filtration rate. For purposes of this discussion, DN is used as a generic term referring to any deleterious effect on kidney structure and/or function caused by diabetes mellitus.

## Management of diabetic nephropathy

World Health Organisation data report age-standardised death rate for diabetics in South Africa is 85 per 100 000 compared with 18 in the USA and six per 100 000 in the UK.^3^ The principal reason for the high mortality rates in South Africa is renal failure as a result of DN. Some 30 to 40% of diabetics develop nephropathy, which is the leading cause of ESRD.^14^

DN progresses through five well-defined stages.^58^ Stage 1 is an increase in GFR, which progresses to the clinically silent stage 2, in which hyperfiltration is associated with hypertrophy. Stage 3, or initial nephropathy, is typified by microalbuminuria, modest increases in blood pressure and a reduction in GFR. Stage 4 sees macroalbuminuria, raised blood pressure and progressive reductions in GFR, leading to stage 5 or ESRD when renal-replacement therapy is required.

ESRD is managed in developed countries by renal replacement therapy (RRT), such as dialysis and transplantation. In developing countries, however, kidney failure rates are double those in the West because access to RRT is severely limited by its high cost to patients.^13^ The figures are stark: 70% of patients in a Nigerian study were able to afford dialysis for only one month, with less than 2% having sufficient resources to remain on dialysis for more than 12 months.^59^ Access to RRT is virtually impossible for the rural poor.^12^

Current conventional diabetes therapy using blood glucose-lowering medications has limitations in averting renal complications. Progression towards ESRD may be slowed in part by strict control of blood sugar levels and blood pressure, a reduction in dietary protein intake and inhibition of the renin–angiotensin system. Consequently, drug developmental strategy should target these metabolic pathways for the prevention of progression to ESRD, which proceeds to DN.

Many patients of sub-Saharan Africa however cannot afford these expensive drugs. Hence there is an urgent need to find affordable treatments which are effective in slowing the progression of DN.

## Medicinal plants in the management of diabetic kidney disease

Ethno-medicinal plants have traditionally been used for the treatment of diabetes and its complications. In fact, current pre-clinical and clinical studies have demonstrated that many have beneficial effects on some processes associated with reduced renal function in experimental animals.^60^-^62^ The active phytochemicals responsible for their activities have also been identified.

**Fig. 1.**
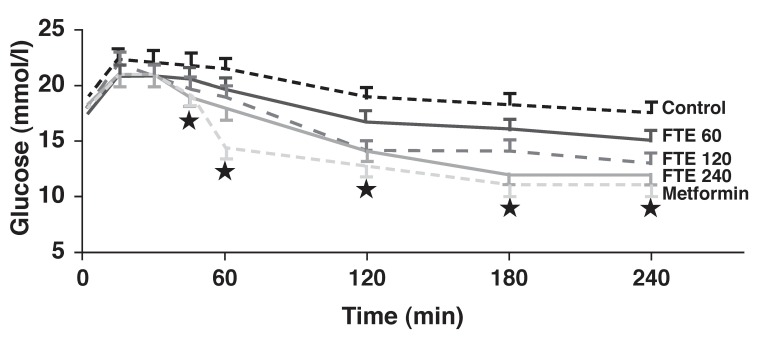
Oral glucose tolerance test in STZ-diabetic rats showing dose-related reduction in plasma glucose levels following treatment with *F thonningii* bark ethanolic extracts (FTE, 60–240 mg/kg) comparable to that induced by metformin (500 mg/kg).^17^ Statistical comparison of the differences between the control and experimental group means was performed using one-way analysis of variance (ANOVA ) followed by Tukey-Kramer multiple comparison test. A value of *p* < 0.05 was considered significant.

Our research has established the therapeutic and pharmacological properties of a number of ethno-botanical herbs traditionally used in the management of diabetes mellitus by African communities.^15^ Observations indicate that some herbal extracts contain compounds that could be effective in mild diabetes mellitus or in cases of impaired glucose tolerance (Fig. 1). These are likely to have a positive impact on glucose homeostasis in diabetic patients.

Investigations from our laboratory have also examined whether herbal extracts could lower blood pressure or improve the impaired renal and cardiovascular functions often seen in diabetes. The results suggest that while some extracts such as *Hypoxis hemerocallidea* corm aqueous extract (APE) had hypoglycaemic effects, they may have deleterious effects on kidney function. Gondwe *et al*. found that APE increased renal fluid output and electrolyte retention, and reduced glomerular filtration rate,^32^ neither of which are desirable in diabetes mellitus. In contrast, other studies from our laboratories have shown that *Opuntia megacantha* leaf extract, which had hypoglycaemic effects, reversed the inability of the kidney to excrete Na^+^ in STZ diabetes mellitus, suggesting that this plant may be beneficial.^17^

**Fig. 2.**
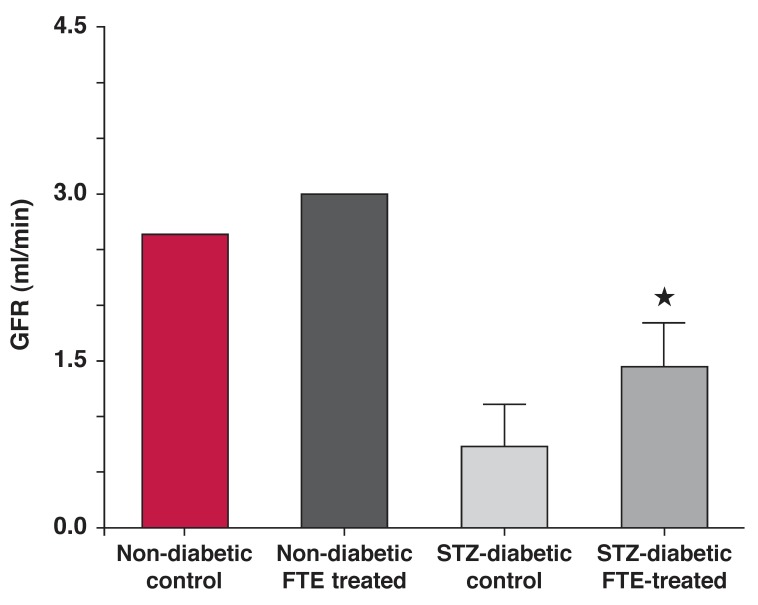
Sub-chronic treatment with *F thonningii* bark ethanolic extracts (FTE) every third day increased glomerular filtration rate in STZ-diabetic rats.^63^

We undertook a systematic survey of medicinal plants used by rural communities in South Africa and have identified several species with beneficial effects in the prevention of renal complications in diabetes mellitus. These effects were observed with both crude extracts and bioactive compounds isolated from antidiabetic plants. In particular, we showed that plants such as *Sclerocarya birrea* [(A Rich) Hochst] [Anachardiaceae], *Persea americana* (Miller) [Lauraceae], *Ficus thonningii* (Blume) [Moraceae] and *Helichrysium ceres* had reno-protective effects (Fig. 2).^17^,^32^,^38^ Initial studies have shown that extracts from these plants ameliorated renal dysfunction in experimental diabetes.

**Fig. 3.**
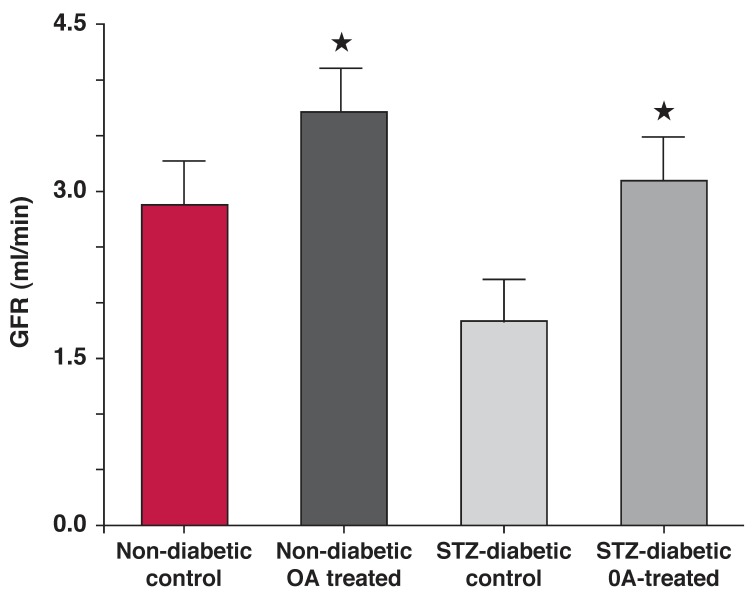
Sub-chronic treatment with oleanolic acid (OA, 60 mg/kg bid every third day) increased glomerular filtration rate in STZ-diabetic rats.^66^

Subsequently, we isolated oleanolic acid as the bioactive compound and have shown that it possesses reno-protective effects in experimental diabetes mellitus. Therefore *S cordatum*-derived oleanolic acid caused increased renal Na^+^ excretion in STZ-induced diabetic rats, which was mediated by an improvement in glomerular filtration rate (Fig. 3).^63^ Other active agents identified in these plants include polysaccharides, flavonoids, xanthones and peptides.

There are various mechanisms by which reno-protection may be achieved, including modulation of AGEs, of the polyol pathway, and of the PKC pathway, and anti-oxidative properties. For example, morroniside isolated from *Corni fructus* has shown reno-protection in experimental diabetes through a reduction in the production of AGEs.^64^ Additionally, some plants have been shown to cause an improvement in renal function in experimental diabetes mellitus through inhibition of ET-1 and TGF-β_1_ and the endothelin-1 receptor A (ETRA).^65^

Available evidence suggests that some herbal extracts interfere with the concentrating and diluting mechanisms of tubular transport processes in the proximal and distal tubules and/or on other components of tubular cell membranes. Therefore we speculate that oleanolic acid influences renal fluid and electrolyte handling by altering the structural integrity and function of tubular epithelial cells to affect reabsorption and secretion.

Modification of risk factors in diabetes has an impressive impact on morbidity and mortality in diabetic patients. An overview of some of some medicinal plants currently used in diabetic hypertension and kidney disease, together with the possible mechanism(s) is summarised in Table 1.

**Table 1.** Partial Survey Of Medicinal Plants/Plant Extracts Which Affected The Cardiovascular And Kidney Function In Diabetes Mellitus.

*Botanical species*	*Bioactive compounds*	*Antidiabetic advantages*	*Renal function advantages*	*Cardiovascular advantages*	*References*
*Allium sativum* L (garlic)	phenols	↑ insulin secretion	↑ GFR	vasorelaxant, ↓ hypolipidaemic	67, 68
(Alliaceae)	flavonoids	↑ hepatic glycogen			
*Gongronema latifolium*	flavonoids	↑ hepatic glycogen	anti-oxidant	↓ hypolipidaemic	69
	saponins				
	polyphenols				
*Foeniculum vulgare* L	phytoestrogens	↓ glucose absorption	diuretic	vasorelaxant	70
(Apiaceae)			natriuretic		
*Opuntia megacantha*	phenols, flavonoids	↓ glucose absorption	↑ GFR	vasorelaxant	71, 72, 73
	(quercetin) taxifolin				
*Syzygium *spp	phenylpropanoids	↑ hepatic glycogen	↑ GFR	vasorelaxant	63, 66, 74
	flavonoids sesquiterpenes	↑ insulin secretion	anti-oxidant		
	oleanolic acid rhamnetin				
*Sclerocarya birrea*	flavonoids,	↑ hepatic glucose	↑ GFR	vasorelaxant	32, 75
[(A Rich) Hochst]	alkaloids, triterpenoids,	utilisation			
[Anacardiaceae]	coumarins, ascorbic acid	↑ insulin secretion			
*Persea americana* Mill	tannins, saponins	↑ hepatic glycogen	↑ GFR	vasorelaxant	32, 76, 77,
(Lauraceae) [Avocado]	flavonoids, alkaloids	↑ insulin secretion		bradycardia	78
	glycosides			↓ hypolipidaemic	
*Hypoxis hemerocallidea*	glycoside hypoxoside	↑ insulin secretion	reno-toxic	cardiodepressant	79, 80
	β-sitosterol sterolins, cytokinins		↓ GFR	bradycardia	
*Ficus thonningii* (Blume)	alkaloids anthraquinones	↑ hepatic glycogen	↑ GFR	cardiodepressant	17, 81
[Morarceae]	flavonoids saponins			vasorelaxant	
	tannins			bradycardia	
*Olea europaea* L,	triterpenes, flavonoids,	↑ insulin secretion	↑ GFR	cardiodepressant	36, 82, 83,
(Oleaceae)	glycosides	↑ glucose utilisation	antioxidant	vasorelaxant	84
				bradycardia	
*Helichrysum ceres* S	polyphenols, tannins,	unclear	diuretic	cardiodepressant	38, 39
Moore	triterpenes		natriuretic	vasorelaxant,	
[Asteraceae]	saponins			bradycardia	
*Ekebergia capensis*	saponins	unclear	unclear	cardiodepressant	85
Sparrm	alkaloids			vasorelaxant	
(Meliaceae)	flavonoids			bradycardia	
	tannins				

## Conclusion

We describe the therapeutic and pharmacological evidence in support of some of the medicinal plant extracts used in the management of hypertension and kidney disease in diabetes mellitus. Some of these medicinal plant extracts are a potential source of anti-diabetic drugs because of their therapeutic efficacy and anti-diabetic mechanisms reported in experimental animals. However, at present, the cellular/molecular mechanisms of action of these plant extracts remain to be established.

Future research directed at the identification of active components is the only viable option for supporting the efficacy claims for all herbs. In the absence of such standardisation, health practitioners and consumers alike should remain optimistic but wary. Research funding to investigate potentially beneficial effects of medicinal plants is critically important for optimal patient care and safety.
